# Supplementary material for: The Medical Genome Reference Bank contains whole genome and phenotype data of 2570 healthy elderly
Source: Nat Commun. 2020 Jan 23;11:435. doi: 10.1038/s41467-019-14079-0 (PMC6978518; doi:10.1038/s41467-019-14079-0)
Supplement: Supplementary file 1 — Supplementary Information [file 41467_2019_14079_MOESM1_ESM.pdf]

Supplementary information for Pinese et al: The Medical Genome Reference Bank contains whole genome and phenotype data of 2,570 healthy elderly

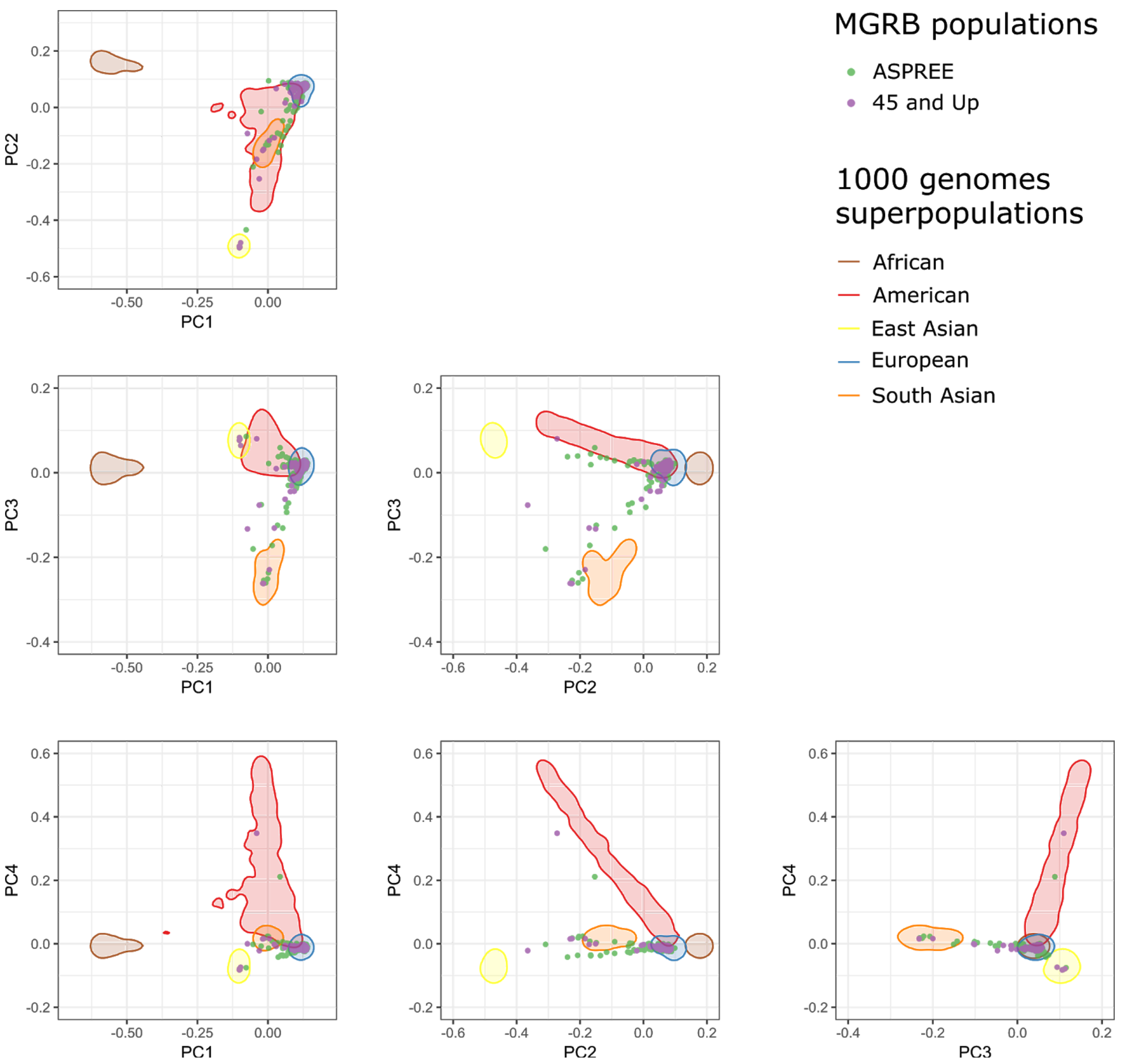

Supplementary Figure 1: Population structure in the MGRB. The MGRB was combined with the 1000 Genomes cohort at high-confidence SNVs, and PCA was performed following LD pruning. Four strong components resulted, scores for which are shown relative to 95% kernel density estimates of the 1000 Genomes superpopulations. The MGRB cohort was largely homogeneous and clustered with the 1000 Genomes European superpopulation.

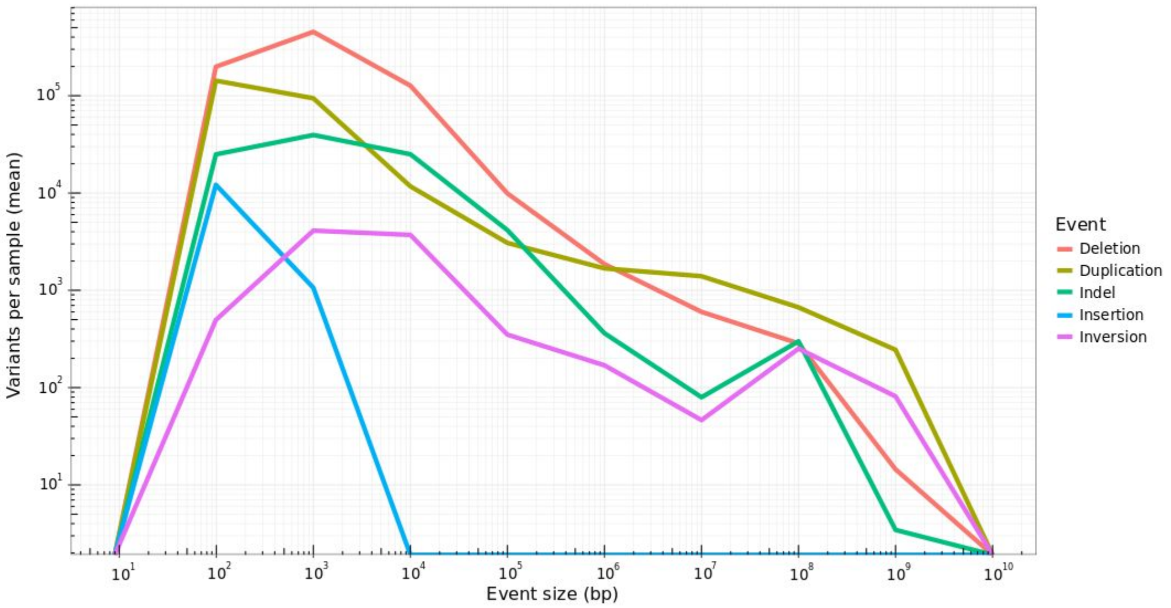

Supplementary Figure 2: Distribution of structural variant event types and sizes detected in the MGRB by GRIDSS.

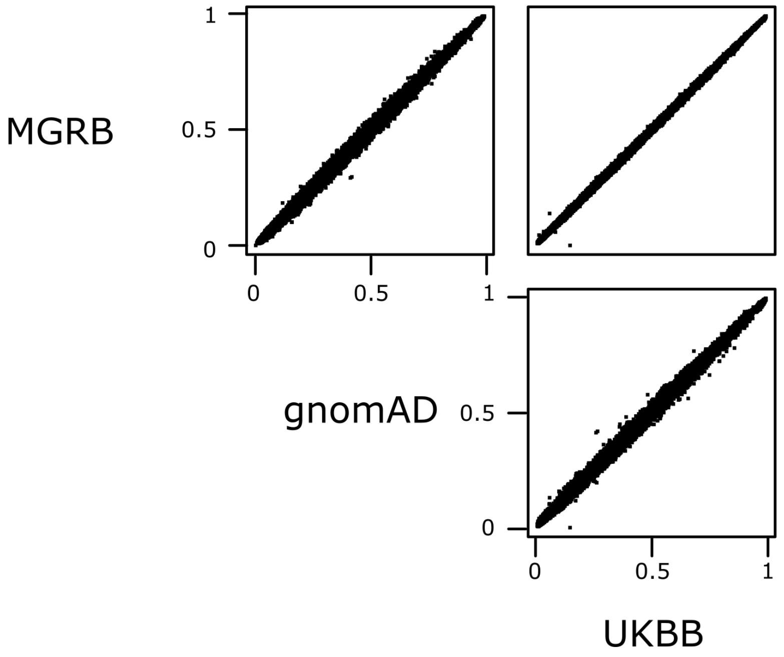

Supplementary Figure 3: SNP alternate allele frequencies compared between MGRB, gnomAD, and UK Biobank cohorts. Only strand-specific biallelic SNPs in well-called regions and reported in the EBI GWAS catalogue are shown.

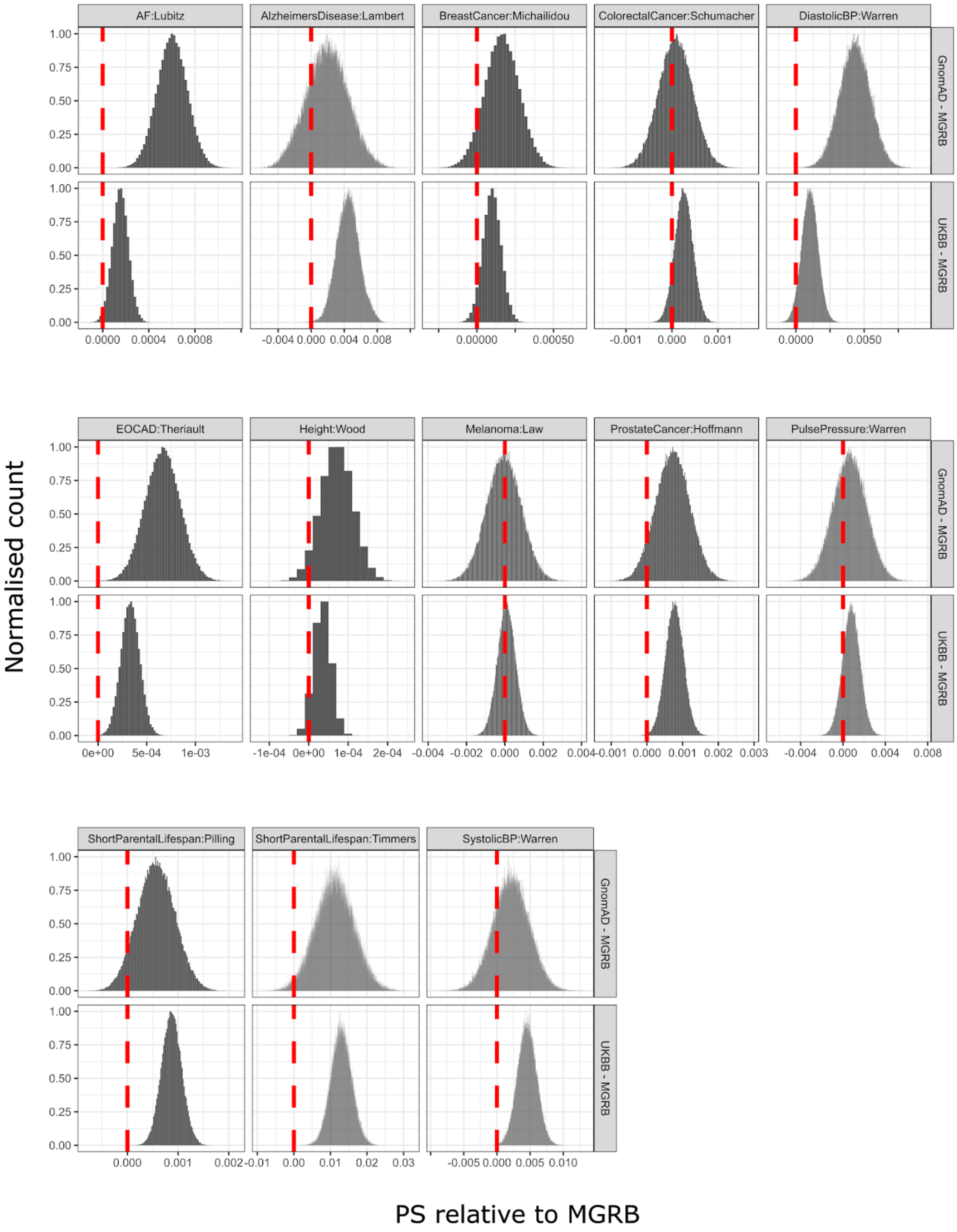

Supplementary Figure 4: Distribution of polygenic score (PS) differences between MGRB and bootstrapped reference cohorts derived from either the GnomAD (top rows), or UK Biobank (bottom rows) baselines. Positive values indicate a PS depletion in MGRB; zero is indicated by dotted red lines. Distributions consistently above zero are indicative of a depletion of PS in MGRB that is not consistent with neutral allele frequency drift between GnomAD / UK Biobank and the Australian population.

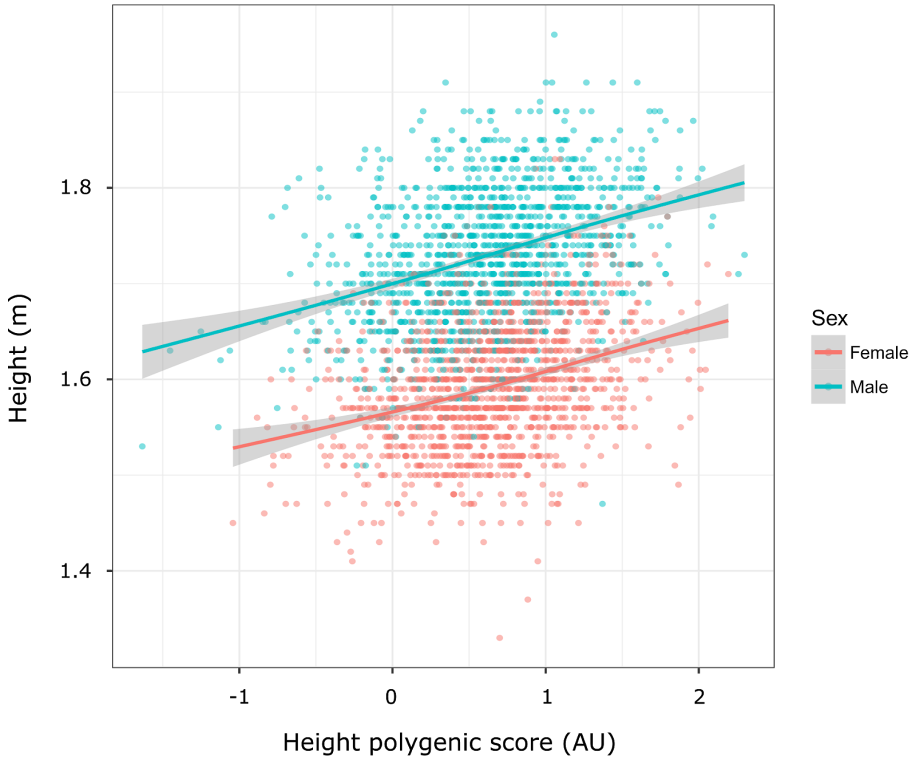

Supplementary Figure 5: Prediction of height in MGRB using a polygenic score [1]. Each point represents the predicted and observed height of an MGRB individual; lines denote GCV-penalised generalised additive model thin plate spline fits, with bands representing 95% confidence intervals for the mean.

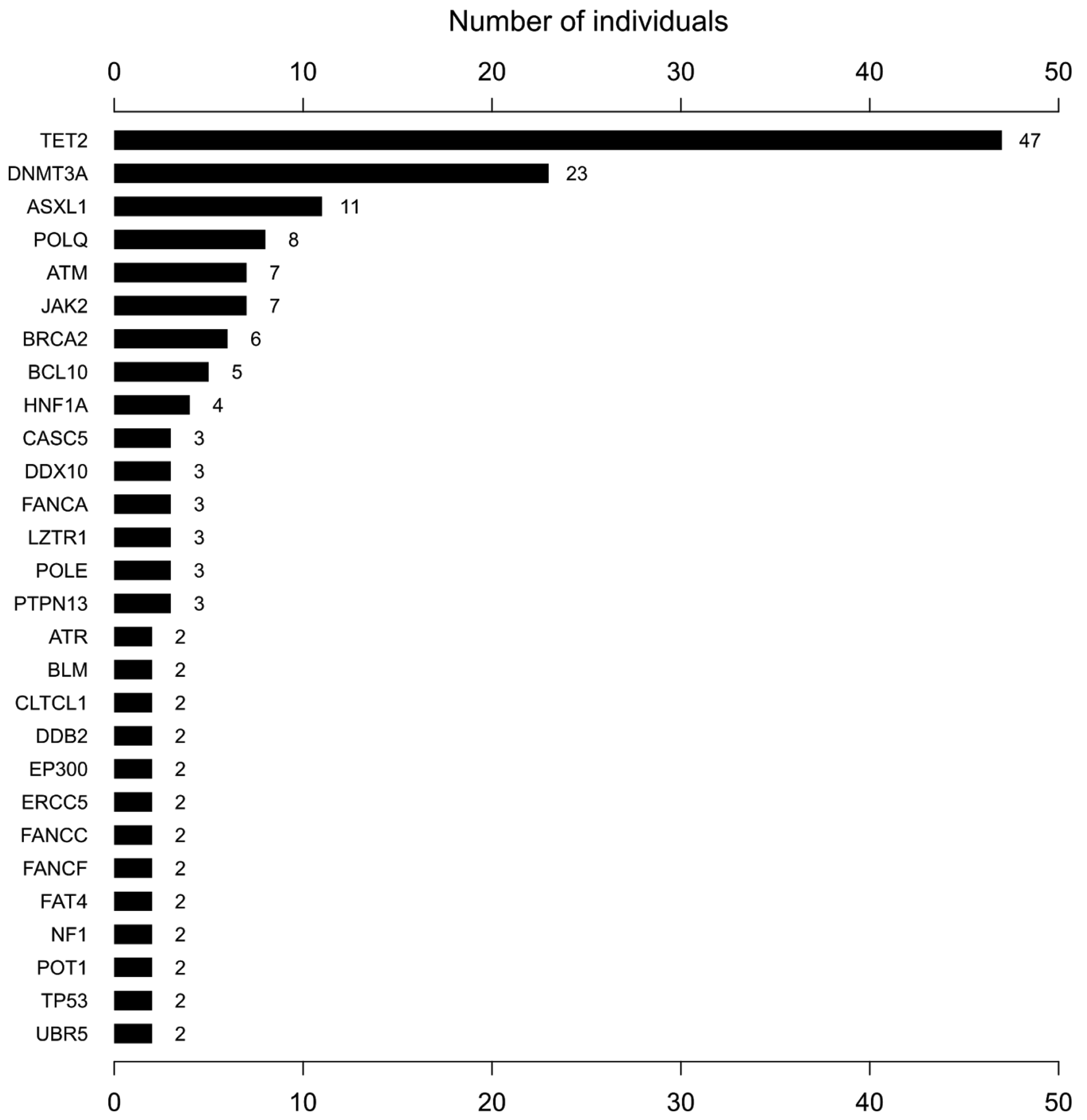

Supplementary Figure 6: Distribution of genes affected by putative subclonal single-nucleotide and indel variation in the MGRB cohort. For each gene, the number of MGRB individuals who harboured a small variant that was either predicted to abrogate function, or coincided with a COSMIC hotspot variant, is shown. 36 genes were affected in only one individual each, and are not displayed.

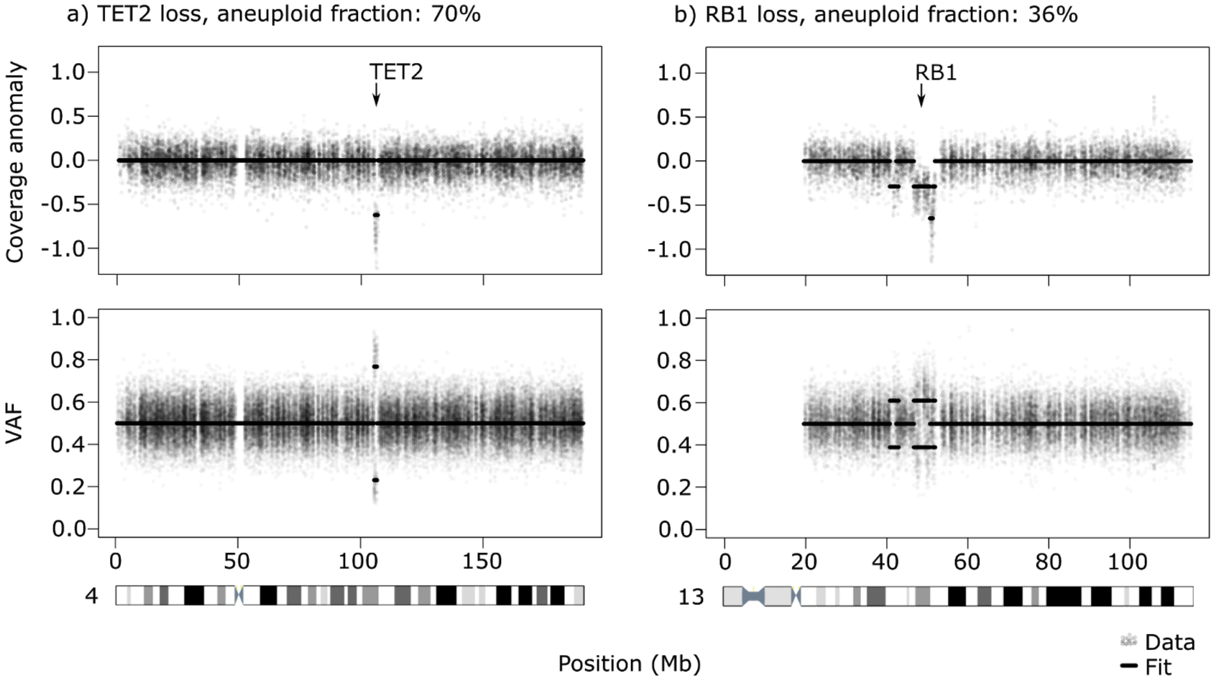

Supplementary Figure 7: Examples of subclonal copy number variation observed in the MGRB. Figures show background-corrected coverage (top panels) and heterozygous variant allele frequency (bottom panels) as a function of genomic location. Individual locus measurements are represented by semi-transparent dots, with model fits indicated by horizontal segments. These samples demonstrated loss of a single copy of TET2 in an estimated 70% of nucleated blood cells (a), or loss of a single copy of RB1 in approximately 36% of cells (b). Coverage is background-corrected and on a $\log_2$ scale, with zero indicating diploidy.

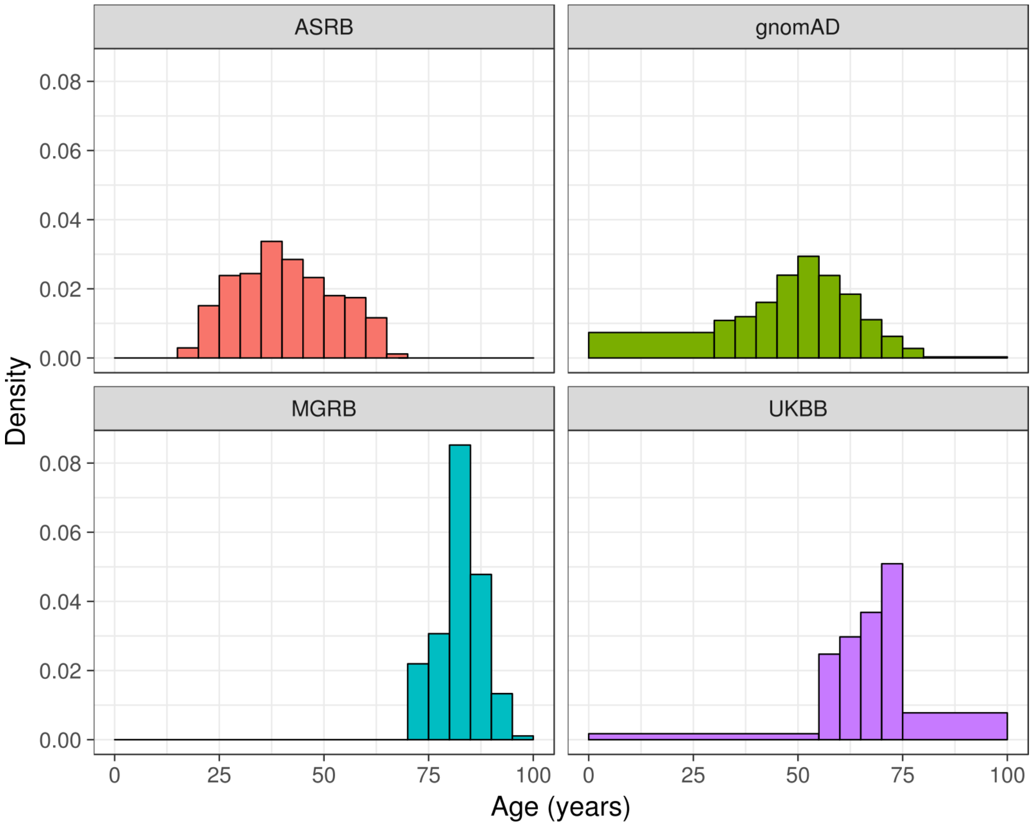

Supplementary Figure 8: Distribution of participant ages in the Australian Schizophrenia Research Bank (ASRB), gnomAD, MGRB, and UK Biobank (UKBB) cohorts. Ages were truncated at 100 years and binned into five year intervals except for terminal bins, which vary in size as shown.

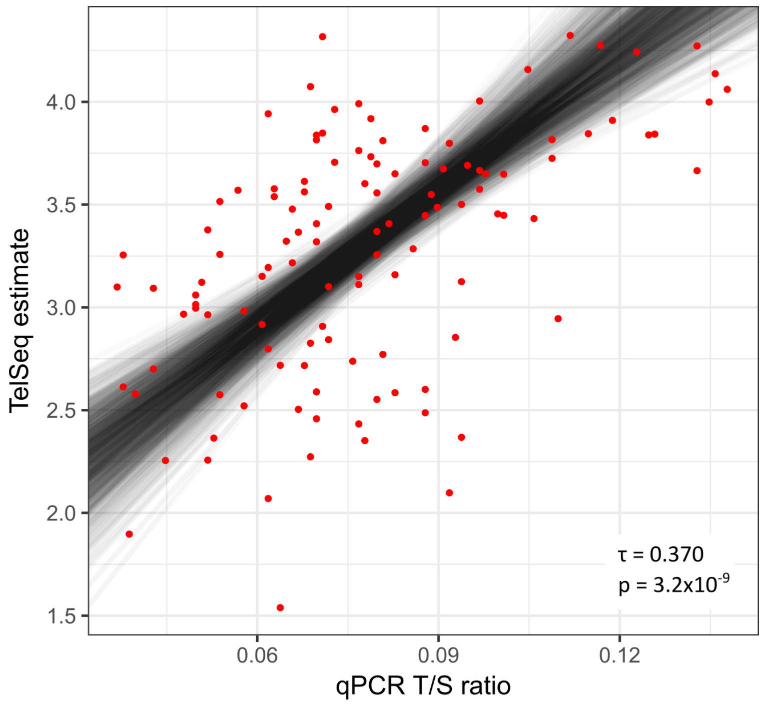

Supplementary Figure 9: Comparison of Telseq WGS telomere length estimates to qPCR measurements. Points denote 119 randomly-selected samples from the MGRB and ASRB cohorts; one outlier with a Telseq estimate over 5 was excluded. Telseq estimates are directly as reported by the software; qPCR measurements are telomere / single copy gene copy ratios. Lines represent fits from 1000 bootstrap replicates of Deming regression using within-bootstrap median absolute deviation as an empirical variance estimate. The measures are significantly correlated (Kendall's $\tau = 0.370$, $p = 3.2 \times 10^{-9}$).

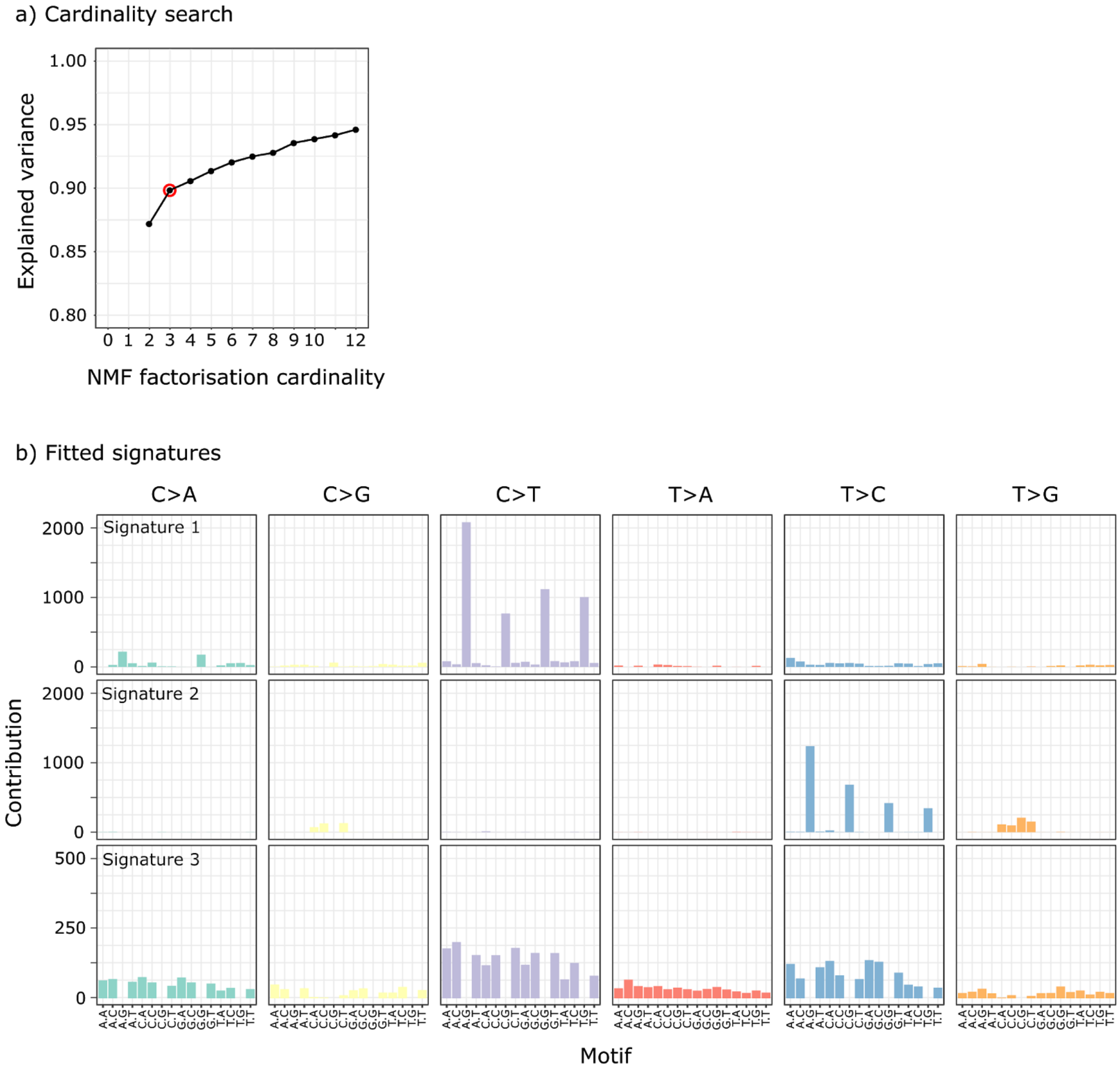

Supplementary Figure 10: Somatic variant motif factorization. A cardinality search on age-grouped samples indicated that a cardinality of 3 was appropriate, being the inflection point on the explained variance vs cardinality plot (a, selected cardinality marked with red circle). When the single-sample motif frequencies were factorized at this selected cardinality, the three signatures resulting were well resolved (b), with Signatures 1 and 3 respectively resembling Signatures 1 and 5 as previously reported [2].

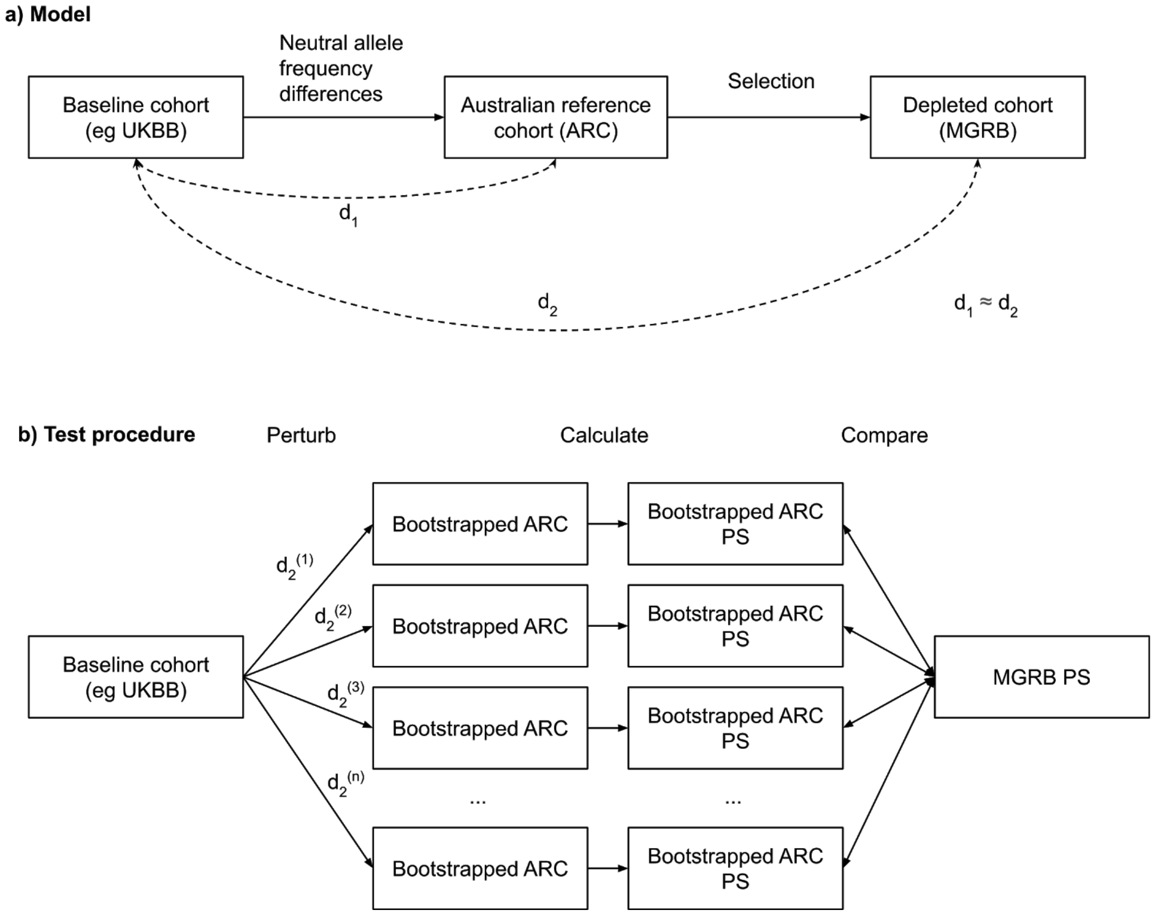

Supplementary Figure 11: Test procedure for polygenic score (PS) differences, accounting for neutral allele frequency differences between populations. The underlying model is that the MGRB is derived by selection from an unobserved Australian reference cohort (ARC), which in turn is derived by neutral allele frequency differences from a baseline cohort such as the UK Biobank (UKBB) (a). As most alleles are unselected, the MGRB-baseline allele frequency differences (d_2) closely approximate the ARC-baseline differences (d_1). This permits the simulation of the unmeasured ARC by derivation from the baseline cohort (b): bootstrap samples of d_2 are used to perturb the baseline allele frequencies and generate a simulated ARC, PS of which are compared to those observed in MGRB. A consistently lower PS in MGRB relative to the bootstrapped ARC PS scores indicate that drift is insufficient to explain the PS depletion in MGRB, and that another factor (eg selection) is likely at play.

Supplementary Table 1: Rates of structural variation (SV) detected in the MGRB. Mean event counts per individual are given, with standard deviation in parentheses.

SV class	*Rate*	*(SD)*	*Fraction*
GRIDSS-reported			
Insertion	45	(7)	0.5%
Deletion	2750	(136)	33.1%
Indel	327	(21)	3.9%
Duplication	882	(98)	10.6%
Inversion	32	(5)	0.4%
Total GRIDSS	4036	(249)	48.6%
Mobster-reported			
L1 insertion	1072	(380)	12.9%
ALU insertion	2754	(246)	33.2%
SVA insertion	436	(92)	5.3%
HERV insertion	3	(2)	< 0.1%
Total Mobster	4264	(634)	51.4%
Grand total	8300	(675)	100%

Supplementary Table 2: Singleton and polymorphic rates for structural variants (SVs) identified in the MGRB. Structural variant count and fraction are given as a function of the number of samples identified to share that variant. Sample ranges are inclusive.

Samples with variant	*SV count (fraction)*	
1 (singletons)	155287	(17.1)
2 - 10	589771	(65.0)
11 - 100	139340	(15.3)
101 - 500	13669	(1.5)
501 - 1000	4623	(0.5)
1001 - 1500	2178	(0.2)
1501 - 2000	1367	(0.2)
2001 - 2500	1218	(0.1)
2501 - 2570	486	(0.1)

Supplementary Table 3: Structural variants identified in MGRB that may disrupt an ACMG incidentally-reportable gene.

Gene	*Variant*	*Predicted effect*
PCSK9	1:g.55494888_55509044del	Loss of 5' UTR and exon 1.
SMAD4	18:g.48556989_48573287del	Loss of 5' UTR.
TMEM43	3:g.141047610_14277101del	Deletion of entire *TMEM43* locus.
VHL	3:g.10067411_10421889inv	Inversion of entire *VHL* locus.

Supplementary Table 4: Clinical and demographic characteristics of the 45 and Up cancer cases, compared to the 45 and Up cancer-free individuals included in the MGRB. Cancer cases had some evidence of a cancer diagnosis prior to age 70, either by self report or admission and registry records; cancer-free individuals had no such evidence prior to age 70. Aggregate statistics are medians, with ranges in parentheses. As some individuals had multiple cancers, the sum of cancer types exceeds the number of cancer cases.

Measure	Cancer cases	Cancer-free
Individuals	269	717
(percent female)	(45.3%)	(59.3%)
Age at collection (years)	71	70
	(64 – 88)	(64 – 91)
Height (m)	1.70	1.66
	(1.47 – 1.96)	(1.37 – 1.91)
Mass (kg)	76.0	72.0
	(44.5 – 120.0)	(36.0 – 147.0)
Cancer type		
Prostate	74	——
Melanoma of skin	58	——
Colorectal	40	——
Breast	26	——
Non-melanoma skin	20	——
Lung	13	——
Bladder	10	——
Other	124	——

Supplementary Table 5: Two-stage testing results for age- and sex-conditioned association between somatic measures and age-related functional changes.

Physical function measure	Somatic measure	First stage p-value		Second stage p-value
		Males, n=240	Females, n=223	Males, n=720
Gait speed	Telomere length	0.952	0.556	—
	Mitochondria number	0.544	0.347	—
	Y copy number	0.501	—	—
	Somatic variant burden	0.605	0.767	—
	Mitochondrial variants	0.941	0.603	—
Grip strength	Telomere length	0.293	0.908	—
	Mitochondria number	0.051	0.732	0.0356
	Y copy number	0.723	—	—
	Somatic variant burden	0.505	0.730	—
	Mitochondrial variants	0.476	0.457	—

Supplementary References

1. Wood, A. R. *et al.* Defining the role of common variation in the genomic and biological architecture of adult human height. *Nat. Genet.* **46**, 1173 (2014).
2. Alexandrov, L. B. *et al.* Clock-like mutational processes in human somatic cells. *Nat. Genet.* **47**, 1402–1407 (2015).
