# Supplementary material for: The Medical Genome Reference Bank contains whole genome and phenotype data of 2570 healthy elderly
Source: Nat Commun. 2020 Jan 23;11:435. doi: 10.1038/s41467-019-14079-0 (PMC6978518; doi:10.1038/s41467-019-14079-0)
Supplement: Supplementary file 5 — Dataset 4 [file 41467_2019_14079_MOESM5_ESM.docx]

## Supplementary Data 1: Resources used

| *Resource* | *Source* | *Identifier* |
| --- | --- | --- |
| TruSeq Nano DNA HT library | Illumina |  |
| HiSeq X clustering and sequencing reagents | Illumina | v2.5 |
| Human reference genome, 1000 Genomes Project phase 3 build 37 with decoy | 1000 Genomes Project | ftp://ftp.1000genomes.ebi.ac.uk/vol1/ftp/technical/reference/human_g1k_v37.fasta.gz |
| ΦX174 genome | NCBI RefSeq | NC_001422.1 |
| Consensus CDS coding regions, accessed 21 Nov 2017 | UCSC browser table ccdsGene | https://genome.ucsc.edu/cgi-bin/hgTables |
| Repetitive regions, last updated 27 April 2009 | UCSC annotations database | http://hgdownload.soe.ucsc.edu/goldenPath/hg19/database/rmsk.txt.gz |
| ENCODE excludable regions, DAC, last updated 5 May 2011 | UCSC browser table wgEncodeDacMapabilityConsensusExcludable | http://hgdownload.soe.ucsc.edu/goldenPath/hg19/database/wgEncodeDacMapabilityConsensusExcludable.txt.gz |
| ENCODE excludable regions, Duke, last updated 29 March 2011 | UCSC browser table wgEncodeDukeMapabilityRegionsExcludable | http://hgdownload.soe.ucsc.edu/goldenPath/hg19/database/wgEncodeDukeMapabilityRegionsExcludable.txt.gz |
| Poor alignment or uniqueness, CRG, last updated 27 April 2010 | UCSC browser table wgEncodeCrgMapabilityAlign100mer score < 1 | https://genome.ucsc.edu/cgi-bin/hgTables |
| Poor alignment or uniqueness, Duke, last updated 12 May 2011 | UCSC browser table wgEncodeDukeMapabilityUniqueness35bp score < 1 | https://genome.ucsc.edu/cgi-bin/hgTables |
| Infinium QC Array 24 1.0 loci | Illumina | https://support.illumina.com/downloads/infinium-qc-array-24-v1-0-support-files.html |
| GiaB HG001 high-confidence regions 3.3.2 | [^1^](https://paperpile.com/c/Et7nLX/ZBzly) | ftp://ftp-trace.ncbi.nlm.nih.gov/giab/ftp/release/NA12878_HG001/latest/GRCh37/HG001_GRCh37_GIAB_highconf_CG-IllFB-IllGATKHC-Ion-10X-SOLID_CHROM1-X_v.3.3.2_highconf_nosomaticdel.bed |
| 1000 Genomes Project phase 3 genotypes, released 2 May 2013 | [^2^](https://paperpile.com/c/Et7nLX/wknfz) | ftp://ftp.1000genomes.ebi.ac.uk/vol1/ftp/release/20130502/ |
| Haplotype reference consortium allele frequencies, 1-1 | [^3^](https://paperpile.com/c/Et7nLX/mDV9H) |  |
| GnomAD allele frequencies 2.0.1 |  | http://gnomad.broadinstitute.org/ |
| dbSNP 150 | [^4^](https://paperpile.com/c/Et7nLX/yiHsC) | http://www.haplotype-reference-consortium.org/ |
| ClinVar, downloaded 9 September 2017 | [^5^](https://paperpile.com/c/Et7nLX/XkjIq) | https://www.ncbi.nlm.nih.gov/clinvar/ |
| CATO 1.1 | [^6^](https://paperpile.com/c/Et7nLX/Wc93d) | http://www.mauranolab.org/CATO/ |
| Eigen coding 1.1 (9 May 2016) | [^7^](https://paperpile.com/c/Et7nLX/S7cMv) | http://www.columbia.edu/~ii2135/eigen.html |
| COSMIC Cancer Gene Census, downloaded 26 April 2018 |  | http://www.sanger.ac.uk/science/data/cancer-gene-census |
| UK Biobank | [^8^](https://paperpile.com/c/Et7nLX/SPhOi) | https://www.ukbiobank.ac.uk/ |
| BWA 0.7.15 |  | https://github.com/lh3/bwa |
| biobambam2 2.0.65-release-20161130121735 | [^9^](https://paperpile.com/c/Et7nLX/kgaQo) | https://github.com/gt1/biobambam2 |
| samtools 1.5 | [^10^](https://paperpile.com/c/Et7nLX/GmGhK) | https://github.com/samtools |
| mdust commit 3e3fed8 |  | https://github.com/lh3/mdust |
| GATK 3.7.0-gcfedb67 | [^11^](https://paperpile.com/c/Et7nLX/6Qqac) | https://software.broadinstitute.org/gatk/ |
| vt 0.5722-60f436c3 | [^12^](https://paperpile.com/c/Et7nLX/SQ09s) | https://github.com/atks/vt |
| Hail 0.1-0320a61 | [^13^](https://paperpile.com/c/Et7nLX/TsHKO) | https://github.com/hail-is/hail |
| VEP 90-3fcc9dd | [^14^](https://paperpile.com/c/Et7nLX/oMYhP) | https://github.com/Ensembl/ensembl-vep |
| TelSeq 0.0.1-be185ec | [^15^](https://paperpile.com/c/Et7nLX/8UCAP) | <https://github.com/zd1/telseq>, tagged release 0.0.1, commit be185ec, downloaded Feb 8, 2017 |
| GRIDSS 1.4.1 | [^16^](https://paperpile.com/c/Et7nLX/Evzjl) | <https://github.com/PapenfussLab/gridss>, version 1.4.1 |
| FreeBayes 1.1.0-54-g49413aa |  | <https://github.com/ekg/freebayes>, version 1.4.1 |
| R 3.5.0 | [^17^](https://paperpile.com/c/Et7nLX/cBXse) | https://www.r-project.org/ |
| GENESIS 2.8.0 | [^18^](https://paperpile.com/c/Et7nLX/ICZe3) | https://bioconductor.org/packages/release/bioc/html/GENESIS.html |
| SNPrelate 1.12.1 | [^19^](https://paperpile.com/c/Et7nLX/b0xPn) | https://bioconductor.org/packages/release/bioc/html/SNPRelate.html |
| mclust 5.3 | [^20^](https://paperpile.com/c/Et7nLX/WLC7i) | https://cran.r-project.org/web/packages/mclust/index.html |
| mgcv 1.8-17 | [^21^](https://paperpile.com/c/Et7nLX/ydeTe) | https://cran.r-project.org/web/packages/mgcv/index.html |
| SomaticSignatures 2.16.0 | [^22^](https://paperpile.com/c/Et7nLX/mlrMV) | https://bioconductor.org/packages/release/bioc/html/SomaticSignatures.html |
| NCI LDLink | [^(Machiela and Chanock 2015)^](https://paperpile.com/c/Et7nLX/fzBf) | <https://ldlink.nci.nih.gov/> |

# References

1. [Zook, J. M. *et al.* Integrating human sequence data sets provides a resource of benchmark SNP and indel genotype calls. *Nat. Biotechnol.* **32**, 246–251 (2014).](http://paperpile.com/b/Et7nLX/ZBzly)

2. [The 1000 Genomes Project Consortium. A global reference for human genetic variation. *Nature* **526**, 68–74 (2015).](http://paperpile.com/b/Et7nLX/wknfz)

3. [McCarthy, S. *et al.* A reference panel of 64,976 haplotypes for genotype imputation. *Nat. Genet.* **48**, 1279–1283 (2016).](http://paperpile.com/b/Et7nLX/mDV9H)

4. [Sherry, S. T. *et al.* dbSNP: the NCBI database of genetic variation. *Nucleic Acids Res.* **29**, 308–311 (2001).](http://paperpile.com/b/Et7nLX/yiHsC)

5. [Landrum, M. J. *et al.* ClinVar: public archive of relationships among sequence variation and human phenotype. *Nucleic Acids Res.* **42**, D980–5 (2014).](http://paperpile.com/b/Et7nLX/XkjIq)

6. [Maurano, M. T. *et al.* Large-scale identification of sequence variants influencing human transcription factor occupancy in vivo. *Nat. Genet.* **47**, 1393–1401 (2015).](http://paperpile.com/b/Et7nLX/Wc93d)

7. [Ionita-Laza, I., McCallum, K., Xu, B. & Buxbaum, J. D. A spectral approach integrating functional genomic annotations for coding and noncoding variants. *Nat. Genet.* **48**, 214–220 (2016).](http://paperpile.com/b/Et7nLX/S7cMv)

8. [Sudlow, C. *et al.* UK biobank: an open access resource for identifying the causes of a wide range of complex diseases of middle and old age. *PLoS Med.* **12**, e1001779 (2015).](http://paperpile.com/b/Et7nLX/SPhOi)

9. [Tischler, G. & Leonard, S. biobambam: tools for read pair collation based algorithms on BAM files. *Source Code Biol. Med.* **9**, 13 (2014).](http://paperpile.com/b/Et7nLX/kgaQo)

10. [Li, H. *et al.* The Sequence Alignment/Map format and SAMtools. *Bioinformatics* **25**, 2078–2079 (2009).](http://paperpile.com/b/Et7nLX/GmGhK)

11. [DePristo, M. A. *et al.* A framework for variation discovery and genotyping using next-generation DNA sequencing data. *Nat. Genet.* **43**, 491–498 (2011).](http://paperpile.com/b/Et7nLX/6Qqac)

12. [Tan, A., Abecasis, G. R. & Kang, H. M. Unified representation of genetic variants. *Bioinformatics* **31**, 2202–2204 (2015).](http://paperpile.com/b/Et7nLX/SQ09s)

13. [Ganna, A. *et al.* Ultra-rare disruptive and damaging mutations influence educational attainment in the general population. *Nat. Neurosci.* **19**, 1563–1565 (2016).](http://paperpile.com/b/Et7nLX/TsHKO)

14. [McLaren, W. *et al.* The Ensembl Variant Effect Predictor. *Genome Biol.* **17**, 122 (2016).](http://paperpile.com/b/Et7nLX/oMYhP)

15. [Ding, Z. *et al.* Estimating telomere length from whole genome sequence data. *Nucleic Acids Res.* **42**, e75 (2014).](http://paperpile.com/b/Et7nLX/8UCAP)

16. [Cameron, D. L. *et al.* GRIDSS: sensitive and specific genomic rearrangement detection using positional de Bruijn graph assembly. *Genome Res.* (2017). doi:](http://paperpile.com/b/Et7nLX/Evzjl)[10.1101/gr.222109.117](http://dx.doi.org/10.1101/gr.222109.117)

17. [R Core Team. R: A Language and Environment for Statistical Computing. (2017). Available at:](http://paperpile.com/b/Et7nLX/cBXse) [https://www.R-project.org/.](https://www.r-project.org/.)

18. [Conomos, M. P. & Thornton, T. GENetic EStimation and Inference in Structured samples (GENESIS): Statistical methods for analyzing genetic data from samples with population structure and/or relatedness. *R package version 2* (2016).](http://paperpile.com/b/Et7nLX/ICZe3)

19. [Zheng, X. *et al.* A high-performance computing toolset for relatedness and principal component analysis of SNP data. *Bioinformatics* **28**, 3326–3328 (2012).](http://paperpile.com/b/Et7nLX/b0xPn)

20. [Scrucca, L., Fop, M., Murphy, T. B. & Raftery, A. E. mclust 5: Clustering, Classification and Density Estimation Using Gaussian Finite Mixture Models. *R J.* **8**, 289–317 (2016).](http://paperpile.com/b/Et7nLX/WLC7i)

21. [Wood, S. N. Stable and Efficient Multiple Smoothing Parameter Estimation for Generalized Additive Models. *J. Am. Stat. Assoc.* **99**, 673–686 (2004).](http://paperpile.com/b/Et7nLX/ydeTe)

22. [Gehring, J. S., Fischer, B., Lawrence, M. & Huber, W. SomaticSignatures: inferring mutational signatures from single-nucleotide variants. *Bioinformatics* **31**, 3673–3675 (2015).](http://paperpile.com/b/Et7nLX/mlrMV)
