# Supplementary material for: The Medical Genome Reference Bank contains whole genome and phenotype data of 2570 healthy elderly
Source: Nat Commun. 2020 Jan 23;11:435. doi: 10.1038/s41467-019-14079-0 (PMC6978518; doi:10.1038/s41467-019-14079-0)
Supplement: Supplementary file 6 — Dataset 5 [file 41467_2019_14079_MOESM6_ESM.pdf]

soma-snv: Background and Derivations

Mark Pinese

December 12, 2019

Consider the problem of identifying somatic SNVs from NGS data. Non-reference bases at a locus may be due to a subclonal SNV, machine error, poorly-sampled heterozygous sites, or mapping artefacts. The primary goal is to efficiently distinguish true subclonal SNV events from these other possibilities, and maintain good SNR even with relatively low sequencing depth. A secondary goal is to quantify the 'callability' of each locus, to permit normalisation of the detected somatic variant rate to the effective number of loci examined.

A simple SNV detector

For simplicity, we consider each locus independently, and aim to only identify somatic variants at homozygous reference germline loci. Let the germline genotype at the locus be $G \in \{\mathrm{RR}, \mathrm{RA}, \mathrm{AA}\}$, where R represents the reference allele and A all possible non-reference alleles, collapsed to a single symbol. Represent the observed allelic read depths by n_R and n_A, and the total depth by $n = n_\mathrm{R} + n_\mathrm{A}$; with corresponding random variables N_R, N_A, and N. Suppose that the sequencing process has a fixed per-base error rate ϵ, where an error means that an incorrect base has been reported by the sequencer (ie in the case of an error, the sequencer will never report the correct base).

Express the tail probability of at least the observed number of A alleles being due to machine error at a homozygous reference locus as

$$\begin{aligned}\Pr(\mathrm{err}) &= \Pr(N_\mathrm{A} \geq n_\mathrm{A} \mid N = n, G = \mathrm{RR}) \\ &= \sum_{i=n_\mathrm{A}}^{n} \mathrm{Binom}(i; n, \epsilon)\end{aligned}$$

and similarly the tail probability that the counts could be due to a poorly-sampled het locus, assuming unbiased allele sampling, as

$$\begin{aligned}\Pr(\mathrm{het}) &= \Pr(N_\mathrm{A} \leq n_\mathrm{A} \mid N = n, G = \mathrm{RA}) \\ &= \sum_{i=0}^{n_\mathrm{A}} \mathrm{Binom}(i; n, \frac{1}{2} + \frac{1}{3}\epsilon)\end{aligned}$$

where

$$\mathrm{Binom}(i; n, p) \equiv \binom{n}{i} p^i (1-p)^{n-i}$$

Given the above, we define a simple SNV caller that reports a subclonal SNV at a locus if the following is satisfied:

$$\mathrm{CALL} \equiv (\Pr(\mathrm{err}) < \alpha_{\mathrm{E}}) \wedge (\Pr(\mathrm{het}) < \alpha_{\mathrm{H}})$$

with α_{E} an error threshold for a false positive due to noise at a reference locus, and α_{H} an error threshold for a false positive due to poor sampling at a heterozygous locus.

Informally, this simple variant caller identifies variants which have too many A alleles to be plausibly due to machine error ($\Pr(\mathrm{err}) < \alpha_{\mathrm{E}}$), yet too few to be plausibly due to a poorly-sampled heterozygous locus ($\Pr(\mathrm{het}) < \alpha_{\mathrm{H}}$).

Adaptive filtering thresholds

The performance of the simple caller described above critically depends on the values of α_{E} and α_{H}. Here we derive a method to determine values for these thresholds that are adaptive to local sequencing depth and user-specified parameters, and achieve optimal sensitivity for a given limit on false positive rate.

Let the rate of germline heterozygous loci be r_{h}, and the fraction of germline homozygous reference (RR) loci with subclonal variation be r_{c}. We proceed to characterise the expected sensitivity p_{n} and SNR p_{r} to detect a subclonal variant of allele frequency f.

Define performance metrics as

$$p_{\mathrm{r}} \equiv \frac{\Pr(\mathrm{SCV} \mid \mathrm{CALL})}{\Pr(\neg\mathrm{SCV} \mid \mathrm{CALL})}$$

$$p_{\mathrm{n}} \equiv \Pr(\mathrm{CALL} \mid \mathrm{SCV})$$

with SCV denoting the true presence of a subclonal variant at the locus.

Expanding the above,

$$\begin{aligned}
\Pr(\mathrm{SCV} \mid \mathrm{CALL}) &= \frac{\Pr(\mathrm{CALL} \mid \mathrm{SCV})\Pr(\mathrm{SCV})}{\Pr(\mathrm{CALL})} \\
&= \frac{\Pr(\mathrm{CALL} \mid \mathrm{SCV}) r_{\mathrm{c}}}{\Pr(\mathrm{CALL})} \\
\Pr(\neg\mathrm{SCV} \mid \mathrm{CALL}) &= \frac{\Pr(\mathrm{CALL} \mid \neg\mathrm{SCV})\Pr(\neg\mathrm{SCV})}{\Pr(\mathrm{CALL})} \\
&= \frac{\Pr(\mathrm{CALL} \mid \neg\mathrm{SCV})(1 - r_{\mathrm{c}})}{\Pr(\mathrm{CALL})} \\
\implies p_r &= \frac{\Pr(\mathrm{CALL} \mid \mathrm{SCV})}{\Pr(\mathrm{CALL} \mid \neg\mathrm{SCV})} \frac{r_{\mathrm{c}}}{1 - r_{\mathrm{c}}}
\end{aligned}$$

The conditional call probability at a true subclonal locus can be simplified

$$\begin{aligned}
\Pr(\mathrm{CALL} \mid \mathrm{SCV}) &= r_{\mathrm{RR}} \ \Pr(\mathrm{CALL} \mid \mathrm{SCV}, G = \mathrm{RR}) \\
&+ r_{\mathrm{RA}} \ \Pr(\mathrm{CALL} \mid \mathrm{SCV}, G = \mathrm{RA}) \\
&+ r_{\mathrm{AA}} \ \Pr(\mathrm{CALL} \mid \mathrm{SCV}, G = \mathrm{AA}) \\
&= r_{\mathrm{RR}} \ \Pr(\mathrm{CALL} \mid \mathrm{SCV}, G = \mathrm{RR})
\end{aligned}$$

with $r_{\mathrm{RA}} = r_{\mathrm{h}}$, $r_{\mathrm{RR}} = \frac{1}{2}\left(1 - r_{\mathrm{h}} + \sqrt{1 - 2r_{\mathrm{h}}}\right)$, and $r_{\mathrm{AA}} = \frac{1}{2}\left(1 - r_{\mathrm{h}} - \sqrt{1 - 2r_{\mathrm{h}}}\right)$ the marginal probabilities of each genotype class, assuming Hardy-Weinberg equilibrium at hetrozygous loci. The second and third terms are zero by construction: we have defined subclonal variants to only exist at homozygous reference loci ($G = \mathrm{RR}$). Note that r_{c} should be chosen to take this restricted definition into account. Similarly,

$$\begin{aligned}
\Pr(\mathrm{CALL} \mid \neg\mathrm{SCV}) &= r_{\mathrm{RR}} \ \Pr(\mathrm{CALL} \mid \neg\mathrm{SCV}, G = \mathrm{RR}) \\
&+ r_{\mathrm{RA}} \ \Pr(\mathrm{CALL} \mid \neg\mathrm{SCV}, G = \mathrm{RA}) \\
&+ r_{\mathrm{AA}} \ \Pr(\mathrm{CALL} \mid \neg\mathrm{SCV}, G = \mathrm{AA}) \\
&\approx r_{\mathrm{RR}}\alpha_{\mathrm{E}} + r_{\mathrm{RA}}\alpha_{\mathrm{H}}
\end{aligned}$$

where the first term equals $r_{\mathrm{RR}}\alpha_{\mathrm{E}}$ and the second $r_{\mathrm{RA}}\alpha_{\mathrm{H}}$ by construction, and the third term is approximately zero (as a $G = \mathrm{AA}$ locus will almost certainly fail the one-sided heterozygous germline locus exclusion filter, and due to the r_{AA} term will be rare besides).

Expanding the conditional true detection probability at a homozygous reference germline locus,

$$\Pr(\mathrm{CALL} \mid \mathrm{SCV}, G = \mathrm{RR}) = \sum_{n_{\mathrm{A}}=0}^{n} \binom{n_{\mathrm{A}}}{n} {p_{\mathrm{A}}}^{n_{\mathrm{A}}} (1 - p_{\mathrm{A}})^{n - n_{\mathrm{A}}} \, [\mathrm{CALL}]$$

with

$$p_{\mathrm{A}} = \left(1 - \frac{4}{3}\epsilon\right) f + \epsilon$$

The Iverson brackets in the above can be removed by observing that the [CALL] term defines bounds on the summation of the n_A allele count distribution: let $c_\mathrm{E} \equiv \inf\{n_\mathrm{A} : \Pr(\mathrm{err}) < \alpha_\mathrm{E}\}$ and $c_\mathrm{H} \equiv \sup\{n_\mathrm{A} : \Pr(\mathrm{het}) < \alpha_\mathrm{H}\}$, then

$$\begin{aligned}
&\mathrm{CALL} \\
\implies &\Pr(\mathrm{err}) < \alpha_\mathrm{E} \wedge \Pr(\mathrm{het}) < \alpha_\mathrm{H} \\
\implies &\sum_{i=n_\mathrm{A}}^{n} \mathrm{Binom}(i; n, \epsilon) < \alpha_\mathrm{E} \wedge \sum_{i=0}^{n_\mathrm{A}} \mathrm{Binom}(i; n, \frac{1}{2}) < \alpha_\mathrm{H} \\
\implies &n_\mathrm{A} \geq c_\mathrm{E} \wedge n_\mathrm{A} \leq c_\mathrm{H} \\
\implies &c_\mathrm{E} \leq n_\mathrm{A} \leq c_\mathrm{H}
\end{aligned}$$

and so

$$\Pr(\mathrm{CALL} \mid \mathrm{SCV}, G = \mathrm{RR}) = \sum_{n_\mathrm{A}=c_\mathrm{E}}^{c_\mathrm{H}} \binom{n}{n_\mathrm{A}} {p_\mathrm{A}}^{n_\mathrm{A}} (1 - p_\mathrm{A})^{n-n_\mathrm{A}}$$

These c_E and c_H are effectively critical values of the respective tests.

Combining the above, we have

$$\begin{aligned}
p_\mathrm{n} &= r_\mathrm{RR} \sum_{n_\mathrm{A}=c_\mathrm{E}}^{c_\mathrm{H}} \binom{n}{n_\mathrm{A}} {p_\mathrm{A}}^{n_\mathrm{A}} (1 - p_\mathrm{A})^{n-n_\mathrm{A}} \\
p_\mathrm{r} &= \frac{r_\mathrm{c}}{1 - r_\mathrm{c}} \frac{p_\mathrm{n}}{r_\mathrm{RR}\alpha_\mathrm{E} + r_\mathrm{RA}\alpha_\mathrm{H}}
\end{aligned}$$

Optimisation

The goal is identify thresholds α_E and α_H that yield optimal sensitivity p_n for a given n and f, subject to SNR p_r being at least as good as a minimum acceptable g_r. This optimisation is performed by the following method:

1. Perform a scan along the $c_\mathrm{E} = c_\mathrm{H}$ axis to identify an initial search starting point $(c_\mathrm{E}, c_\mathrm{H})$ where $p_\mathrm{r} \geq g_\mathrm{r}$.

2. Assess p_r and p_n at candidate points $(c_\mathrm{E} - 1, c_\mathrm{H})$, $(c_\mathrm{E}, c_\mathrm{H} - 1)$, and $(c_\mathrm{E} - 1, c_\mathrm{H} - 1)$. If at least one of the candidate points have $p_\mathrm{r} \geq g_\mathrm{r}$ and has improved p_n, update the current point to this best point and repeat. Else, terminate.

As f is a fixed user-specified parameter and n takes on relatively few values, a lookup table containing previously-identified optima makes this simple strategy computationally efficient.

Power and callability

The p_{r} values yielded by the optimisation algorithm above represent estimates of per-sample and locus sensitivity to detect subclonal variants. These p_{r} values can be summed across loci in a sample, possibly subset by sequence context, to yield a measure of total callability for that sample. This measure is a suitable normalisation constant to derive per-sample subclonal SNV rates.

Input data and parameters

Given the simplicity of the error model, only high quality data should be used. In particular, read mapping quality and base quality thresholds are encouraged, and problematic loci (eg non-unique mapping) should be excluded. Preliminary work uses thresholds of MQ ≥ 40, BQ ≥ 30, and excludes poor mapping or low complexity regions.

Reasonable parameters for the caller operating on high-depth sequence data ($>$ 100X) are $r_{\mathrm{h}} = 10^{-3}$, $r_{\mathrm{c}} = 10^{-6}$, $\epsilon = 10^{-2}$, $g_{\mathrm{r}} = 10$. These must be relaxed for more typical data ($\approx$ 30X depth), or else no $(c_{\mathrm{E}}, c_{\mathrm{H}})$ pair will satisfy $p_{\mathrm{r}} \geq g_{\mathrm{r}}$, and no loci will be assessable for somatic variation. The following parameters have been tested on single-lane WGS data (30-40X): $r_{\mathrm{h}} = 10^{-4}$, $r_{\mathrm{c}} = 5 \times 10^{-7}$, $\epsilon = 2 \times 10^{-3}$, $g_{\mathrm{r}} = 5$, $f = 0.2$, although additional variant filtering will be required.
