# Supplementary material for: The Medical Genome Reference Bank contains whole genome and phenotype data of 2570 healthy elderly
Source: Nat Commun. 2020 Jan 23;11:435. doi: 10.1038/s41467-019-14079-0 (PMC6978518; doi:10.1038/s41467-019-14079-0)
Supplement: Supplementary file 8 — Reporting Summary [file 41467_2019_14079_MOESM8_ESM.pdf]

nature research

Corresponding author(s): David M Thomas

Last updated by author(s): Dec 12, 2019

Reporting Summary

Nature Research wishes to improve the reproducibility of the work that we publish. This form provides structure for consistency and transparency in reporting. For further information on Nature Research policies, see Authors & Referees and the Editorial Policy Checklist .

Statistics

For all statistical analyses, confirm that the following items are present in the figure legend, table legend, main text, or Methods section.

n/a	Confirmed	
☐	☒	The exact sample size (*n*) for each experimental group/condition, given as a discrete number and unit of measurement
☐	☒	A statement on whether measurements were taken from distinct samples or whether the same sample was measured repeatedly
☐	☒	The statistical test(s) used AND whether they are one- or two-sided<br>*Only common tests should be described solely by name; describe more complex techniques in the Methods section.*
☐	☒	A description of all covariates tested
☐	☒	A description of any assumptions or corrections, such as tests of normality and adjustment for multiple comparisons
☐	☒	A full description of the statistical parameters including central tendency (e.g. means) or other basic estimates (e.g. regression coefficient) AND variation (e.g. standard deviation) or associated estimates of uncertainty (e.g. confidence intervals)
☐	☒	For null hypothesis testing, the test statistic (e.g. *F*, *t*, *r*) with confidence intervals, effect sizes, degrees of freedom and *P* value noted<br>*Give P values as exact values whenever suitable.*
☒	☐	For Bayesian analysis, information on the choice of priors and Markov chain Monte Carlo settings
☒	☐	For hierarchical and complex designs, identification of the appropriate level for tests and full reporting of outcomes
☒	☐	Estimates of effect sizes (e.g. Cohen's *d*, Pearson's *r*), indicating how they were calculated

Our web collection on statistics for biologists contains articles on many of the points above.

Software and code

Policy information about availability of computer code

Data collection	N/A
Data analysis	All software are open source, with versions given in the Methods and Supplementary Data 1.

For manuscripts utilizing custom algorithms or software that are central to the research but not yet described in published literature, software must be made available to editors/reviewers. We strongly encourage code deposition in a community repository (e.g. GitHub). See the Nature Research guidelines for submitting code & software for further information.

Data

Policy information about availability of data

All manuscripts must include a data availability statement. This statement should provide the following information, where applicable:

- Accession codes, unique identifiers, or web links for publicly available datasets
- A list of figures that have associated raw data
- A description of any restrictions on data availability

Summary variant frequency data for the MGRB cohort are available at the web portal: https://sgc.garvan.org.au/explore. Raw genomic data have been deposited at the European Genome-Phenome Archive under study ID EGAS00001003511 [https://ega-archive.org/studies/EGAS00001003511]. Phenotype data are available upon application to the MGRB Data Access Committee at mgrb@garvan.org.au.

Field-specific reporting

Please select the one below that is the best fit for your research. If you are not sure, read the appropriate sections before making your selection.

Life sciences study design

All studies must disclose on these points even when the disclosure is negative.

Sample size	Sample size was pre-determined based on availability of samples from the contributing cohorts, and budget.
Data exclusions	Some individuals were excluded from the analysed data based on failing quality control thresholds determined during pilot work. These procedures and thresholds are detailed in the methods.
Replication	Reproducibility concerns were addressed by multiple test correction or holdout validation, as appropriate. Details of the methods used in each case are given in the main text and methods.
Randomization	This was a retrospective observational study: experimental manipulations were not performed, so randomization into experimental groups was not required.
Blinding	During rare variant pathogenicity assessment, the clinical geneticist responsible was blinded to health state of the study participants.

Reporting for specific materials, systems and methods

We require information from authors about some types of materials, experimental systems and methods used in many studies. Here, indicate whether each material, system or method listed is relevant to your study. If you are not sure if a list item applies to your research, read the appropriate section before selecting a response.

Materials & experimental systems

n/a	Involved in the study	
☒	☐	Antibodies
☒	☐	Eukaryotic cell lines
☒	☐	Palaeontology
☒	☐	Animals and other organisms
☐	☒	Human research participants
☒	☐	Clinical data

Methods

n/a	Involved in the study	
☒	☐	ChIP-seq
☒	☐	Flow cytometry
☒	☐	MRI-based neuroimaging

Human research participants

Policy information about studies involving human research participants

Population characteristics	This is a major focus of the manuscript and described in detail in the main text.
Recruitment	Participants were recruited by the ASPREE and the 45 and Up Studies, with some comparator control samples sourced from the Australian Schizophrenia Research Bank. It is likely that a healthy cohort effect was present in both groups, however given the design of the study (the deliberate selection of healthy individuals), this will not have caused unwanted bias.<br><br>As an Australian cohort of healthy elderly individuals, the genetic background of the sequenced participants reflects the cultural makeup of early 20th century Australia, and is not representative of a modern Australian population. This will have caused some bias in common allele frequencies, which has been recognised in the text.
Ethics oversight	The study was approved by the following ethics boards:<br>* Monash University Human Research Ethics Committee (ASPREE participants)<br>* Alfred Hospital Ethics Committee (ASPREE participants)<br>* University of New South Wales Human Research Ethics Committee (45 and Up participants)<br>* NSW Population & Health Services Research Ethics Committee (45 and Up participants)<br>* University of Newcastle Human Ethics Research Committee (ASRB comparator controls)

Note that full information on the approval of the study protocol must also be provided in the manuscript.
