# Supplementary material for: The Medical Genome Reference Bank contains whole genome and phenotype data of 2570 healthy elderly
Source: Nat Commun. 2020 Jan 23;11:435. doi: 10.1038/s41467-019-14079-0 (PMC6978518; doi:10.1038/s41467-019-14079-0)
Supplement: Supplementary file 9 — Description of Additional Supplementary Files [file 41467_2019_14079_MOESM9_ESM.pdf]

Title: Supplementary Data 1:
Description: Details of all rare variants curated to be pathogenic or likely pathogenic in ACMG SF 2.0 genes in the MGRB cohort.

Title: Supplementary Data 2:
Description: Common variant score analysis supplementary data.

Title: Supplementary Data 3:
Description: Putative incidental somatic variants identified in the MGRB.

Title: Supplementary Data 4:
Description: Resources used in this work.

Title: Supplementary Data 5:
Description: Development and derivation of the somatic SNV detector.
